# Persistence and risk factors of high-risk human papillomavirus infection among HIV positive and HIV negative tanzanian women: a cohort study

**DOI:** 10.1186/s13027-022-00442-2

**Published:** 2022-06-11

**Authors:** Patricia Swai, Vibeke Rasch, Ditte S. Linde, Bariki Mchome, Rachel Manongi, Chun Sen Wu, Marianne Waldstrom, Thomas Iftner, Julius Mwaiselage, Susanne K. Kjaer

**Affiliations:** 1 Kilimanjaro Christian Medical Centre, 3060 Moshi, Tanzania; 2 University of Southern Denmark, Odense, Denmark; 3 Department of Gynaecology and Obstetrics, Odense University Hospital, Odense, Denmark; 4 Department of Pathology, Lillebaelt Hospital, Vejle, Denmark; 5 Institute of Medical Virology, University Hospital of Tübingen, Tübingen, Germany; 6 Ocean Road Cancer Institute, Dar es Salaam, Tanzania; 7 Danish Cancer Society Research Center, Unit of Virus, Lifestyle and Genes, Copenhagen, Denmark; 8 Department of Gynecology, Rigshospitalet, University of Copenhagen, Copenhagen, Denmark

**Keywords:** HR HPV, Persistence, Risk factors, HIV status, Tanzania

## Abstract

**Background:**

High-risk (HR) human papillomavirus (HPV) persistence is the most important risk factor for cervical cancer. We have assessed the type-specific HR HPV persistence among HIV positive and HIV negative Tanzanian women and factors associated with HR HPV persistence.

**Methods:**

In a cohort study including 4080 Tanzanian women, 3074 attended follow-up examination (up to 32 months after enrollment). Cervical samples were obtained for liquid-based cytology and HPV DNA testing using Hybrid Capture 2 and Inno-Lipa Extra II. Information on lifestyle factors was collected through a personal interview. The probability of HR HPV persistence at a given time point since enrollment was estimated non-parametrically using the EMICM algorithm.

**Results:**

Among the 462 women HR HPV positive at enrollment, 158 had at least one identical type detected at follow-up. The probability of persistence at 18 months after enrollment was 34.2 (95% CI 29.0–39.4). Stratifying by HIV status, the persistence probability was 42.9% (95% CI 33.5–51.9) among HIV positive, and 28.0% (95% CI 22.1–34.2) among HIV negative. Overall, HR HPV persistence was most common for HPV58, 35, 16, 31, and 52. Among HIV positive women it was HPV45, and HPV16, followed by HPV58 and HPV18, and among HIV negative women it was HPV31, HPV33 and HPV58. Risk factors associated with persistence of HR HPV were older age, longer interval between enrollment and follow-up, binge drinking, and HIV status.

**Conclusions:**

HR HPV persistence was common in Tanzania, and most common among HIV positive women. Overall, persistence was most frequent for HPV 58, 35, 16, 31 and 52. The nonavalent HPV vaccine should be considered.

## Introduction

Human papillomavirus (HPV) infection is the most common sexually transmitted infection, and it is associated with virtually all cervical cancer cases [1, 2]. There exist more than 200 HPV genotypes, of which 13 types have been defined as types associated with cancer or probably associated with cancer by the International Agency for Research on Cancer (IARC) (group 1 and 2A), i.e. HPV 16, 18, 31, 33, 35, 39, 45, 51, 52, 56, 58, 59, 68 (high-risk (HR) types). A small fraction of HPV infections persist and can progress to precancerous lesions that may further progress to cancer. It has been suggested that persistence may be influenced by a number of viral, host, and environmental factors, such as HPV type, multiple HPV infections, viral load, infection with human immunodeficiency virus (HIV), older age, and smoking [2, 4].

The highest incidence of cervical cancer is found in Sub-Saharan Africa, especially in Southern and Eastern Africa, which in 2018 had an age-standardized incidence rate of 43.1 and 40.1 per 100,000 women, respectively [5]. Research suggests that even though HPV16 is universally the most common type, some HPV types such as HPV52 and HPV35 may be more prevalent in Sub-Saharan Africa than in Europe [6]. However, relatively little is known about the natural history of HPV in an African context where HIV positivity is more common than e.g. in Europe. Therefore, longitudinal studies are important in these populations in order to identify the women most at risk of persistent infection. However, only few cohort studies on specifically type-specific HR HPV persistence have been performed in East- and Southern Africa, and they are generally limited in size or have been conducted in selected populations or among younger women [7–10]. The aim of this study was to assess persistence of HR HPV and specific HR HPV types in a cohort of Tanzanian women. In addition, we examined the association of different factors with persistence with special attention to the impact of HIV, and the role of other factors when considering HIV status.

## Methods

### Study setting

This study is part of the Comprehensive Cervical Cancer Prevention in Tanzania (CONCEPT) study. A detailed description of the cohort profile has been published elsewhere [11]. Data were collected between August 2015 and October 2018 at two cervical cancer screening clinics in the Kilimanjaro Region; Mawenzi Regional Hospital and Kilimanjaro Christian Medical Centre (KCMC), and one screening clinic in the Dar es Salaam Region; Ocean Road Cancer Institute (ORCI). In the Kilimanjaro region, women from the urban and rural district of Moshi were included, and in the Dar es Salaam region, women from Ilala, Temeke, and Mwananyamala district were included. The cervical cancer screening program has existed in Tanzania since 2011, and it uses public announcement for inclusion to screening and primarily targets women aged 30–50 years [12]. Screening is performed by means of Visual Inspection using Acetic Acid (VIA). A positive VIA result implies treatment with cryotherapy or LEEP (loop electrosurgical excision procedure) depending on the size of the lesion. Cryotherapy is available at all levels of health facilities (dispensaries, health centers and hospitals), while LEEP is available at zonal and regional hospitals only (Ocean Road and KCMC).

### Study population

The study population was recruited from women aged 25–60 years who attended cervical cancer screening. Exclusion criteria were history of total hysterectomy and being pregnant. Women included in the study were tested for HIV, except those already known to be HIV positive. All women had a gynecological examination performed by qualified health care providers where cervical samples were obtained for liquid based cytology and HPV DNA testing. This was followed by VIA according to the Tanzanian cervical cancer screening guidelines [13]. Finally, all women went through a personal interview conducted by health care providers, who had been trained for this task prior to the study start.

At the end of the enrollment visit, all women were invited to attend a follow-up visit after around 14 months. Women who did not return within approximately one month of the scheduled visit were contacted by phone by a community nurse. Women, who did not show up within two weeks after the phone call, had a nurse home-visit encouraging them to visit the clinic. If a woman still did not attend but consented to it, an outreach community nurse visited her again and conducted the follow-up visit at home, where the cervical specimen was obtained by self-sampling. Among women attending the follow-up examination, 72% attended 4–19 months after enrolment, and 28% attended 20–35 months after the first examination.

### HPV testing and genotyping

Cervical cell swabs were collected using a plastic spatula and endocervical brush (Digene, Gaithersburg, MD) and kept in PreservCyt Transport Medium (Preservcyt Inti Solution Kit, Hologic®) or using a self-sampling kit (Evalyn brush (Rovers Medical Devices)). All samples were shipped to Institute of Medical Virology for analysis (Tuebingen, Germany). The specimens were tested for the presence of HPV DNA using the Hybrid Capture 2 (HC2) test (Qiagen, Hildesheim Germany) with a HR cocktail probe which enables detection of at least 13 HR HPV types, namely 16, 18, 31, 33, 35, 39, 45, 51, 52, 56, 58, 59, 68. We used the United States Food and Drug Administration approved threshold of 1.0 pg HPV DNA/ml, which corresponds to 1.0 relative light unit coefficient. HPV genotyping was performed on all HC2 positive samples using the LiPA Extra II test, which is a line blot assay based on reverse hybridization principles [14]. It is designed to detect 28 HR HPV types (HPV16, 18, 26, 31, 33, 35, 39, 45, 51, 52, 53, 56, 58, 59, 66, 68, 73, 82) as well as 10 low-risk HPV types (6, 11, 40, 43, 44, 54, 70, 69, 71, 74). Specific sequences of the L1 region of the HPV genome were amplified using SPF10 primer pairs and the resulting biotinylated amplicons were then denatured and hybridized with specific oligonucleotide probes. A set of primers for the amplification of the HLA-DPB1 gene was added to monitor sample quality and extraction. Finally, the automated reading of the HPV genotype result for LiPA HPV was provided with the LiRas prototype software.

### HIV status

A blood sample from the index finger was obtained and tested using the HIV-1/2 test (www.alere.com). As confirmation of a positive result, a supplementary quick HIV-1/2 test (Abbott Laboratories) was performed. In case of discordant results, further testing was performed using Unigold (Trinity Biotech). All newly diagnosed HIV patients were referred to the HIV care and treatment clinic for follow-up and treatment according to the Tanzanian national HIV/AIDS guidelines. Further, information on CD4 counts performed within the last 6 months prior to enrolment was obtained from HIV care and treatment (CTC) cards. If this was not possible, attempts were made to obtain the information from the medical records at the CTC clinic.

### Statistical analysis

HR HPV persistence was defined as being positive for the same HR HPV types at enrollment and at follow-up. HR HPV types were defined as types associated with cancer or probably associated with cancer by IARC (group 1 and 2A) (HPV 16, 18, 31, 33, 35, 39, 45, 51, 52, 56, 58, 59, 68) [3].

The probability of HR HPV persistence at a given time since enrollment, that is one minus the probability of having cleared all HR HPV types present at baseline, was estimated non-parametrically using the EMICM algorithm [15] as implemented in PROC ICLIFETEST in SAS. For those observed to have cleared at follow-up, clearance was assumed to have happened somewhere in between enrollment and follow-up (interval-censored); whereas for those still positive for at least one of the HR HPV types observed at baseline, clearance was assumed to happen after the time of follow-up (right censored). Estimation was done overall and according to age group (< / > 40 years) and among women without high-grade intraepithelial lesions or worse (HSIL +). We also estimated the probability of persistence at 18 months after enrollment according to age and HIV status. Similarly, the probability of persistence at 18 months was assessed individually for each HR HPV type overall and according to HIV status.

Further, we assessed factors associated with HR HPV persistence by means of multiple logistic regression analysis, estimating odds ratio (OR) and 95% CI. Adjustment was always made for time between enrolment visit and follow-up visit and age. Subsequently, we additionally adjusted for HIV status and cervical sampling method. The examined factors included socio-demographic factors (age, educational level, marital status), factors related to sampling (time between enrolment and follow-up visit, cervical sampling method), lifestyle factors (binge drinking, i.e. drinking > 6 drinks at the same occasion) and reproductive factors (number of pregnancies, lifetime number of sexual partners), viral factors (HIV status, CD4 count within 6 month of enrolment, and number of HR HPV types at enrolment). We selected these factors a priori based on the literature and availability. These analyses were performed using Stata IC Version 16.1 (Copyright 1985–2017 StataCorp LLC).

## Results

**Fig. 1:**
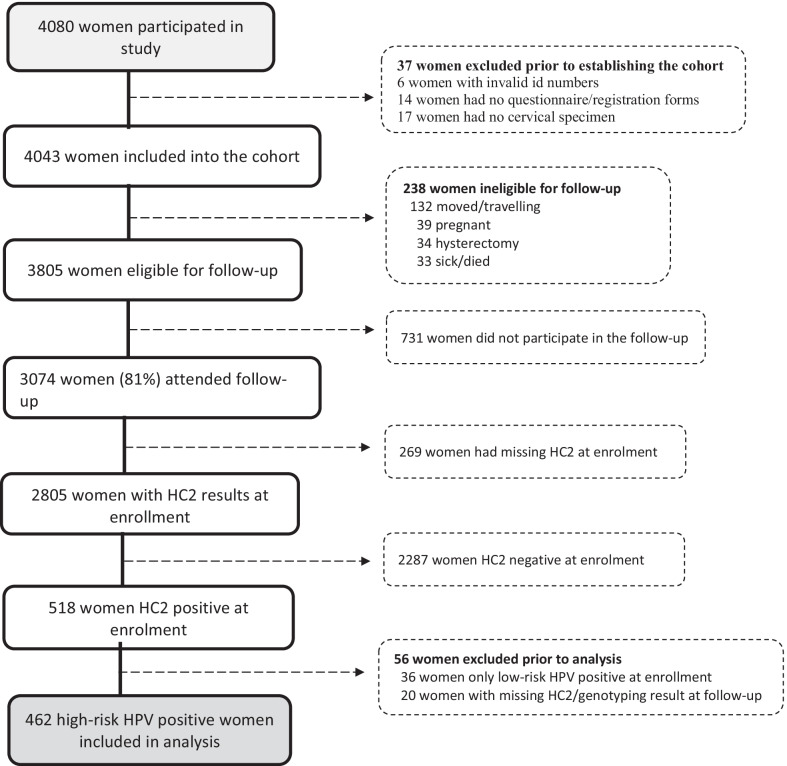
Flow chart describing enrollment and follow-up in a longitudinal study of women from Tanzania

Altogether 4080 were enrolled in the study, and 4043 women were included in the study baseline population. Among these, 3805 women were eligible for follow-up and 3074 women (81%) attended the follow-up examination. Prior to analysis, we excluded 269 women with missing HC2 results at enrollment, leaving a potential study population of 2805 women. Among these, 518 women were HC2 positive at enrollment, yet we excluded 36 women with low-risk HPV only and 20 women with missing HC2 or genotyping result at follow-up, leaving 462 HR HPV positive women for analysis (Fig. 1).

The mean age of the women in the study population was 39 years. Most women had primary education (71.0%), were married/cohabiting (58.7%) and had 3–4 pregnancies (40.7%). The most prevalent HR HPV types at enrollment were HPV52 (22.7%) and HPV16 (19.3%). At enrollment, 45% of the women had multiple HPV types detected.

**Fig. 2:**
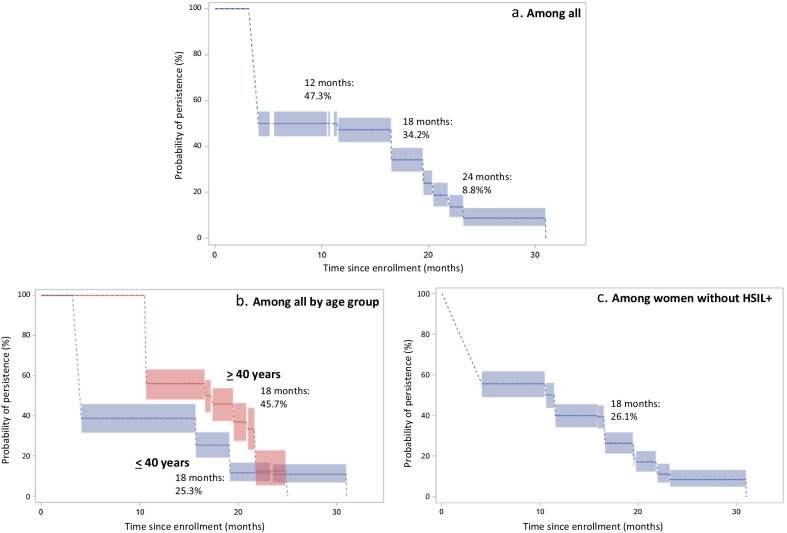
Probability of high-risk (HR) human papillomavirus (HPV) persistence according to time since enrollment among Tanzanian women (N = 462)-overall (**a**), according to age (**b**), and among women without HSIL + (**c**)

Among the 462 women HR HPV positive at enrollment, 158 had the same type at follow-up. The probability of persistence 12 months after enrollment was 47.3% (95% CI 41.9–52.5) (Fig. 2a). The corresponding probabilities at 18 months and 24 months after the first examination were 34.2% (95% CI 29.0–39.4) and 8.8% (95% CI 5.4–13.2), respectively. When stratified for HIV status, the 18 months probability for HR persistence was 28.0% (95% CI 22.1–34.2) among HIV negative women and 42.9% (95% CI 33.5–51.9) among HIV positive women (data not shown). The probability of HR HPV persistence at 18 months among younger women (< 40 years of age) was 25.3% (95% CI 20.9–31.8) and 45.7% (95% CI 37.4–53.7) in women 40 years and older (Fig. 2b). When we restricted the study population to women without HSIL + at enrollment, the overall probability of persistence at 18 months was 26.1% (95% CI 19.8–32.4) and 8.1 (95% CI 4.8–12.9) at 24 months (Fig. 2c). Stratification on HIV status in this restricted population gave a probability of HPV persistence at 18 months of 24.3% (95% CI 18.4–30.6) and 33.3% (95% CI 23.7–43.3) in HIV negative and HIV positive women, respectively (data not shown).

**Fig. 3:**
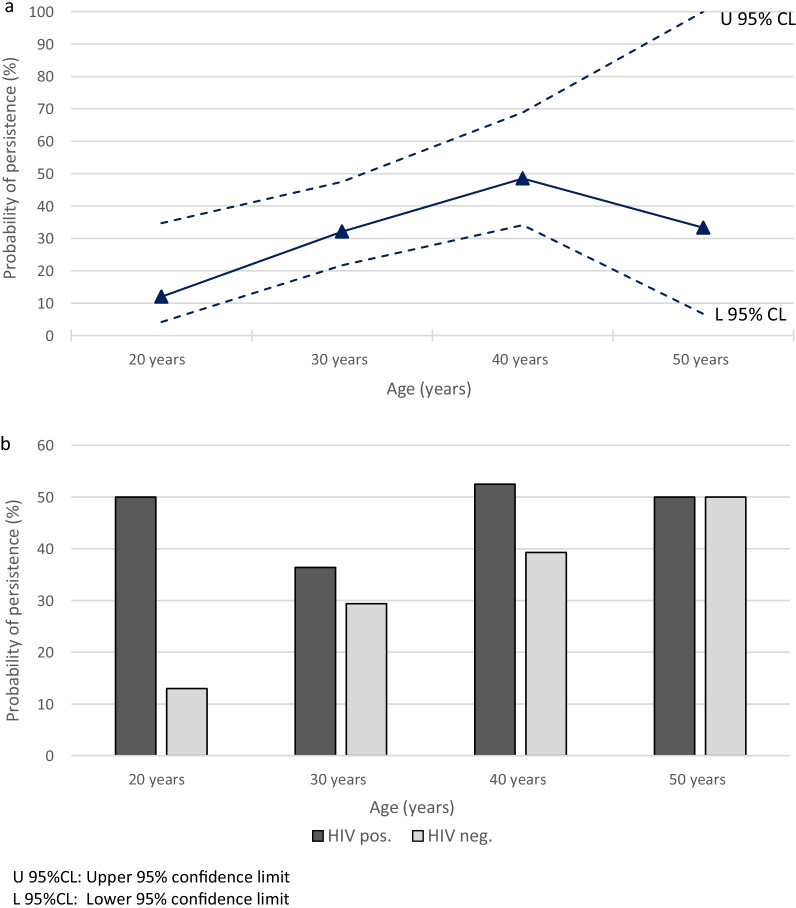
Probability of high-risk (HR) human papillomavirus (HPV) persistence at 18 months after baseline in relation to age among Tanzanian women(N = 462)-overall (**a**) and according to HIV status (**b**)

In Fig. 3, the relationship between persistence and age is displayed in more detail. The overall HR HPV persistence probability at 18 months after enrollment increased with increasing age up until around 40 years and thereafter it stabilized (Fig. 3a). The same pattern was seen both among HIV negative women whereas and among HIV positive women the persistence probability was high in all age groups, and consistently higher than among HIV negative women in all age groups except among the oldest women (Fig. 3b).

Table 1 shows HPV the probability of persistence at 18 months after the first examination for the different HR HPV types. Overall, HR HPV persistence was most common for HPV58, 35, 16, 31, and 52. Among HIV positive women the highest persistence rate was seen for HPV45 and HPV16 followed by HPV58 and HPV18, while in HIV negative women persistence was most frequent for HPV31 by HPV33 and HPV 58.

**Table 1:** Probability of specific high-risk (HR) human papillomavirus (HPV) types persistence at 18 months after baseline among Tanzanian women-overall and according to HIV status

HPV type at enrollment	n/N^a^	Probability of persistence of specific HR HPV types at 18 months after baseline					
		Overall		According to HIV status			
		Probability	(95% CI)	HIV positive		HIV negative	
				Probability	(95% CI)	Probability	(95% CI)
HPV 16	31/89	34.8	(19.9–60.9)	55.6	(31.0–99.7)	30.4	(16.4–56.5)
HPV 18	14/67	25.0	(7.5–83.0)	41.7	(21.4–81.4)	22.7	(10.5–49.1)
HPV 31	20/54	30.0	(11.6–77.3)	33.3	(6.7–100.0)	33.3	(10.8–100.0)
HPV 33	11/29	16.7	(2.8–99.7)	0		20.0	(3.5–100.0)
HPV 35	19/66	37.5	(22.4–62.9)	33.3	(6.7–100.0)	33.3	(16.3–68.2)
HPV 39	4/26	0		0		0	
HPV 45	8/39	0		57.1	(30.1–100.0)	0	
HPV 51	9/57	18.2	(7.5–44.1)	14.3	(2.3–87.7)	18.5	(8.4–40.9)
HPV 52	37/105	29.6	(16.6–53.0)	33.3	(15.0–74.2)	33.3	(15.0–74.2)
HPV 56	8/34	26.7	(11.5–61.7)	40.0	(21.5–74.3)	15.4	(4.3–55.0)
HPV 58	23/70	50.0	(36.1–69.3)	50.0	(30.6–81.6)	37.5	(19.9–70.6)
HPV 59	3/21	12.5	(2.0–78.2)	0		20.0	(5.8–69.1)
HPV 68	11/50	26.9	(14.3–50.7)	33.3	(15.0–74.2)	33.3	(10.8–100.0)

^a^Will add up to more than total due to individuals with multiple types

The associations between different factors and HR HPV persistence are shown in Table 2. Increasing age and shorter time interval between the two examinations turned out to be the strongest risk factors. When comparing a time interval of 12–15 months between enrollment and follow-up with an interval of 20–34 months, the OR was 2.8 (95% CI 1.5–5.5). The OR of persistence was respectively 3.7 (95% CI 1.8–7.6) and 4.0 (95% CI 1.7–9.0) for women aged 40–49 and 50–60 years compared to younger women (25–29 years) after adjustment for time interval between the two examinations, HIV status, and cervical sampling method. In addition, the cervical sampling method influenced the odds of persistence. When we compared self-collected samples with those taken by a health provider, the OR was 0.4 (95% CI 0.2–0.7). Other demographic and reproductive/sexual behavior characteristics like educational level, marital status, and number of sexual partners were not significantly associated with HR HPV persistence, whereas there was a tendency towards increasing odds of persistence with increasing number of pregnancies.

**Table 2:** Association between selected factors and persistence of high-risk (HR) human papillomavirus (HPV) among Tanzanian women (N = 462)

Variable^c^	Total no	% with HR HPV persistence	Adj. OR^a^	(95% CI)	Adj. OR^b^	(95% CI)
Socio-demographic factors						
Age (years)						
25–29	77	(15.6)	1		1	
30–39	174	(28.2)	2.0	(1.0–4.1)	1.9	(0.9–4.0)
40–49	144	(45.8)	4.1	(2.0–8.4)	3.7	(1.8–7.6)
50–60	67	(46.3)	4.1	(1.9–9.2)	4.0	(1.7–9.0)
Educational level						
Primary school	328	(35.7)	1		1	
Secondary school	134	(30.6)	0.9	(0.5–1.4)	0.9	(0.5–1.4)
Marital status						
Married/cohabiting	271	(35.1)	1		1	
Single	190	(33.2)	0.8	(0.6–1.3)	0.7	(0.5–1.1)
*Factors related to sampling*						
No. of months between enrollment and follow-up						
< 12	20	(45.0)	4.5	(1.6–12.6)	3.0	(1.0–8.9)
12–15	151	(47.0)	4.5	(2.5–8.2)	2.8	(1.5–5.5)
16–19	164	(36.0)	3.0	(1.7–5.5)	2.6	(1.4–4.7)
≥ 20	127	(15.0)	1		1	
Sampling method						
Health provider	243	47.4	1		1	
Self-collected	197	18.8	0.4	(0.2–0.7)	0.4	(0.2–0.7)
Method not registered	22	27.3	0.4	(0.1–1.0)	0.4	(0.1–1.0)
*Lifestyle factors*						
Binge drinking^c^						
Never drinking	259	(26.3)	1		1	
Never binge drinking	79	(41.8)	1.6	(0.9–2.8)	1.5	(0.9–2.7)
Ever	121	(47.1)	2.3	(1.4–3.6)	2.2	(1.4–3.7)
No. of pregnancies						
Never pregnant	28	(14.3)	0.6	(0.2–1.8)	0.6	(0.2–1.9)
1–2	159	(25.2)	1		1	
3–4	188	(40.3)	1.6	(1.0–2.6)	1.6	(1.0–2.7)
≥ 5	87	(43.7)	1.5	(0.8–2.8)	1.6	(0.9–3.1)
Lifetime no. of sex partners						
≤ 1	124	(32.3)	1		1	
2	121	(38.8)	1.7	(1.0–3.1)	1.7	(0.9–3.0)
3	94	(35.1)	1.6	(0.9–3.0)	1.5	(0.8–2.9)
≥ 4	117	(29.1)	1.0	(0.6–1.8)	0.9	(0.5–1.7)
*Infections*						
HIV status						
HIV neg	313	(30.0)	1		1	
HIV pos	149	(43.0)	1.4	(0.9–2.2)	1.4	(1.0–2.1)
CD4 count						
HIV neg	313	(30.0)	1		1	
HIV pos./missing CD4	35	(37.1)	1.4	(0.6–2.9)	1.3	(0.6–2.9)
≥ 500	55	(43.6)	1.3	(0.7–2.4)	1.2	(0.7–2.3)
200–499	50	(44.0)	1.5	(0.8–2.9)	1.5	(0.8–2.8)
≤ 199	9	(55.6)	2.3	(0.6–9.3)	2.2	(0.5–9.2)
Number of HPV types						
Single HR HPV	254	(31.5)	1		1	
Multiple HR HPV	208	(37.5)	1.5	(1.0–2.2)	1.4	(0.9–2.1)

^a^Adjusted for age (continuous variable) and time between enrollment and follow-up (continuous variable)

^b^Adjusted for age (continuous variable), time between enrollment and follow-up (continuous variable), HIV-status and sampling method

^c^Defined as more than 6 drinks on the same occasion

We found that alcohol intake increased the odds of HR HPV persistence. Compared to never drinking, binge drinking was associated with increased odds of persistence with a corresponding OR of 2.2 (95% CI 1.4–3.7). HIV infection also increased the odds of persistence when adjusting for age, time between enrollment and follow-up, and sampling method (OR = 1.4; 95% CI 1.0–2.1). The odds also increased with decreasing level of CD4 count (OR = 2.2; 95% CI 0.5–9.2), although statistical significance was not achieved. Finally, we found that women who had two or more HPV types detected had higher odds of persistence than women with a single HR HPV type (OR = 1.4; 95% CI 0.9–2.1).

## Discussion

In this cohort of 462 HR HPV positive Tanzanian women, type-specific HPV persistence was common, and more frequent among HIV positive than HIV negative women. HR HPV persistence at 18 months after enrollment was most common for HPV58, 35, 16, 31, and 52. Increasing age, binge drinking, shorter time interval between HPV samples, HPV sampling method, and HIV status including lower CD4 count, were associated with increased odds of type-specific HR HPV persistence.

We found an overall persistence probability of 34.2% among all women at 18 months after enrollment (42.9% among HIV positive and 28.0% among HIV negative). In comparison, a study from Rwanda including 100 HIV negative women and 137 HIV positive women, found a persistence rate of around 18% (HIV negative) and 59% (HIV positive) (data extracted from Kaplan–Meier curve) at 18 months [10], and another recent study from Rwanda reported a HR HPV persistence of 39.6% after a median follow-up time of 22 months [16]. Similar to our results, Kelly et al. [17] found HPV to be persistent at 18 months in 44.7% of HIV positive women from South Africa and somewhat higher in women from Burkina Faso (55.5%). Demarco et al. [18] reported that in a large study from Kaiser Permanente, Northern California, US, approximately 80% of HPV infections that did not progress to high-grade cervical lesions, cleared within three years. This is in line with our results showing that less than 10% of women without HSIL + had persistence at month 24 after enrollment. Persistence among African populations seems to be high. However, it is challenging to compare results regarding persistence between studies as there is no universally accepted definition of persistence. Time between visits and number of visits vary substantially, and different characteristics of study populations such as age and HIV prevalence together with different HPV detections methods make comparisons of persistence difficult.

Overall, HR HPV persistence was most common for HPV58, 35, 16, 31, and 52, yet differing between HIV positive and HIV negative women. HPV45, and HPV16 followed by HPV58 and HPV18 were the most commonly persistent types among HIV positives while HPV31, 33, and 16 were the most persistent among HIV negatives. However, the results are based on small numbers, and this precludes any firm conclusions. Some differences exist in the literature in relation to findings of which HPV types are most persistent in different populations. Adebamowo et al. [19] reported that HPV52, 35, and 31 were the most persistent HPV types in HIV positive women from Nigeria whereas among HIV positive women from Rwanda, HPV16, 33, and 18 were most persistent [16]. These differences may be caused by differing type-specific sensitivities of the various HPV test used or may reflect that most studies – including our present study – are based on limited numbers when studying persistence of the specific HPV types. Nonetheless, the observed persistence pattern might help inform HPV vaccination strategies in Sub-Saharan Africa. Currently, there are three licensed HPV vaccines: the bivalent targeting HPV16/18, the quadrivalent, which also target HPV6 and HPV11, and the nonavalent which additionally targets HPV types 31, 33, 45, 52, and 58 [2]. The quadrivalent HPV vaccination has since 2018 been provided to Tanzanian schoolgirls of 9–14 years. However, given the persistence patterns found in our study, we suggest that implementation of the nonavalent HPV vaccine should be considered in Tanzania. When discussing vaccination strategies and scale-up of HPV vaccination initiatives, a recent systematic review has highlighted that the level of knowledge on HPV infection and cervical cancer and the level of knowledge of the safety and effectiveness of HPV vaccination are low in Sub-Saharan Africa [20]. In addition, poorly developed adolescent and school health services and poor infrastructures are considered significant challenges for an effective scale-up of HPV vaccination in this area [21]. Moreover, financial constraints due to limited health budgets and high burden of diseases prevail [22]. Altogether, these contextual and financial issues comprise a significant hindrance for the scale-up of HPV vaccination in Tanzania as well as in other Sub-Saharan African countries. Even though global partners, such as GAVI, have made substantial investments and co-financed commitments to make HPV vaccine affordable for many Sub-Saharan African countries [23], international commitment is greatly needed to further develop current HPV vaccine programs.

In studies of risk factors for HPV persistence, it has been suggested that both infections and host factors play a role for persistence of an HPV infection [24]. In line with our results, several studies identified HIV positivity and decreasing CD4 count as important factors, pointing to the importance of the immune system in HPV persistence [10, 16, 19]. We also found a significant association between increasing age and persistent HPV infection. This may indicate an impairment of the immune system with increasing aging, which also has been reported elsewhere [25]. Further, we found that binge drinking increased the odds of HPV persistence, and this finding is supported by other studies [26]. Finally, when assessing viral characteristics of the HPV infections, we found that women, who had two or more HR HPV types at enrollment, had higher odds of persistence than those with one HPV type, although this association did not reach statistical significance. Others have reported similar findings [27]. Potentially this may also be related to a different immune status in women with multiple HPV types [28].

In addition to infection and host factors, we also found an association between sampling factors and HPV persistence, i.e. increased time span between enrollment and follow-up examination was related to decreased HPV persistence. This finding is the line with several previous studies [16, 29]. Further, women who had performed HPV self-sampling had reduced odds of HPV persistence compared to those who had a health provider collected sample taken. HPV self-sampling is a relatively new concept and to our knowledge, no previous studies have reported on this. It may be speculated if the lower HPV persistence found in women who self-sampled vaginal specimens at home reflect that the sampling was not performed optimally, yielding insufficient material for adequate HPV DNA detection. This assumption may be supported by a recent qualitative study, reporting that women preferred the presence of a nurse to help guide them in the sampling procedure as they were insecure in performing the self-sample [30]. Hence, a key aspect for successful implementation of HPV self-sampling in this context involves proper support of the women during the self-sampling.

Our study has several strengths, including a relatively large sample size, broad age range of the study population, high follow-up rate, and long follow-up period as well as comprehensive data on risk factors, and the use of sensitive and well-validated HPV tests for the detection of HPV DNA and HPV genotyping. Additionally, we were able to include a large number of HIV positive women, which allowed for estimating overall and type-specific HPV persistence rate according to HIV status with reasonable statistical power. The main limitation of our study is that despite of a large study population, the numbers of HPV positive woman were still limited. Further, follow-up was challenging, which resulted in a relatively large number of cervical specimens being obtained by self-sampling. This may have resulted in a number of samples being misclassified with regard to the HPV result. Moreover, some of the questions on sexual behaviour and drinking habits were potentially sensitive. Consequently, reporting bias may have hampered our ability to adequately adjust for these factors and residual confounding cannot be excluded. Finally, it would have been relevant to assess the association between HPV persistence and other HIV-related factors such as ART, duration of ART, and HIV viral load. However, we were not able to retrieve detailed information on these factors.

## Conclusion

HR HPV persistence was common but the majority of infections cleared after 24 months. Persistence was more common among HIV positive than HIV negative women. Overall, persistence was most frequent for HPV58, 35, 16, 31, and 52. HIV status, and especially low CD4 count, younger age, binge drinking, time interval between HPV samplings, and HPV sampling method were associated with increased risk of HR HPV persistence. To address the cervical cancer burden in Tanzania, HPV vaccination with the nonavalent vaccine should be recommended. Such vaccination strategy should go hand in hand with well-functioning HPV-based cervical cancer screening programs with special focus on HIV positive women.

## Data Availability

Data and clinical files are available from the corresponding author and investigators from participating Institutions.
